# 919. Disparities in Psychological Trauma After Severe Burns: Insights from a National Cohort

**DOI:** 10.1093/jbcr/irag033.444

**Published:** 2026-04-06

**Authors:** Maria-Meg D Epino, Sarah Salama, Grace S Saglimbeni, Paul Kang, Manuel Cevallos

**Affiliations:** Creighton University School of Medicine, Phoenix, AZ; Creighton University School of Medicine, Phoenix, AZ; Creighton University School of Medicine, Phoenix, AZ; Creighton University School of Medicine, Phoenix, AZ; Creighton University School of Medicine, Phoenix, AZ

## Abstract

**Introduction:**

Burn injuries carry long-lasting psychological consequences, yet large-scale investigation of post-burn trauma remain scarce. Psychological trauma following third-degree burns is often under recognized, particularly across age, sex, and burn location. We seek to investigate the prevalence and independent predictors of psychological trauma among adult third-degree burn patients using a national dataset.

**Methods:**

Using the Nationwide Readmissions Database and ICD-10 codes from 2016-2021, we conducted a secondary analysis of the psychological outcomes of third-degree burn patients. Patients were stratified by sex, burn characteristics, and psychosocial outcomes. Multivariable logistic regression was performed to evaluate predictors of psychological trauma diagnoses, adjusting for burn severity, total body surface area (TBSA), comorbidity index, and hospital characteristics.

**Results:**

Among 38 706 burn patients, 3.6% developed psychological trauma. Prevalence was slightly higher in rural compared with urban patients (3.9% vs. 3.5%; OR 1.23, 95% CI 0.99–1.53). Female sex (OR 1.60, 95% CI 1.34–1.91) and younger age were strong predictors, while older patients had significantly lower odds. Burn site was important, with genital/buttocks (OR 0.66, 95% CI 0.49–0.89) and torso burns (OR 0.79, 95% CI 0.63–0.97) associated with increased trauma risk. Patients with ≥50% TBSA had more than double the odds (OR 2.34, 95% CI 1.36–4.00). Interaction models revealed that rural female patients with genital/buttocks burns were at the greatest risk.

**Conclusions:**

Psychological trauma is a measurable and underrecognized complication of third-degree burns, disproportionately affecting younger patients, women, and those with genital or torso involvement. Rural women with genital burns represent a particularly vulnerable subgroup, reflecting the combined effects of sex, injury location, and geographic disparities. These findings highlight critical gaps in post-burn mental health services and underscore the urgent need for proactive psychological screening, particularly among high-risk populations like rural women.

**Applicability of Research to Practice:**

These findings emphasize the need for routine psychological screening and early referral pathways as part of burn recovery protocols. Incorporating mental health services into standard care may improve long-term outcomes, particularly for high-risk subgroups defined by sex, geography, and burn severity.

**Funding for the study:**

Mark and Jason Yamaguchi Endowment.

